# Association between Serum Albumin Concentration and Ketosis Risk in Hospitalized Individuals with Type 2 Diabetes Mellitus

**DOI:** 10.1155/2016/1269706

**Published:** 2016-07-18

**Authors:** Po-Chung Cheng, Shang-Ren Hsu, Yun-Chung Cheng

**Affiliations:** ^1^Division of Endocrinology and Metabolism, Department of Internal Medicine, Changhua Christian Hospital, 135 Nanxiao Street, Changhua City, Changhua County 500, Taiwan; ^2^Department of Radiology, Taichung Veterans General Hospital, 1650 Taiwan Boulevard Sector 4, Taichung 40705, Taiwan

## Abstract

*Objective.* This study examined the association between serum albumin concentration and ketosis risk in hospitalized individuals with type 2 diabetes mellitus (T2DM).* Methods*. A retrospective cross-sectional study was conducted at a medical center in Taiwan. Inclusion criteria were endocrinology ward inpatients exceeding 21 years of age, with preexisting diagnosis of T2DM, and blood glucose above 13.9 millimoles per liter (mmol/L) at admission. Individuals without measurement of serum albumin, urine ketone, or hemoglobin A1C, or harboring active infection, myocardial infarction, cerebrovascular event, cirrhosis, malignancy, or overt proteinuria were excluded. Using serum albumin concentration below 3.0 grams per deciliter to define hypoalbuminemia, 151 hypoalbuminemic cases and 104 normoalbuminemic controls were enrolled. The presence of ketones in urine established ketosis.* Results*. The prevalence of ketonuria was 48% in hypoalbuminemic subjects compared to 30% in normoalbuminemic controls (odds ratio (OR): 2.15; 95% confidence interval (CI): 1.26–3.57; *P* = 0.004). Moreover, among the 156 subjects with serum beta-hydroxybutyrate measurement in addition to urine ketone, 33% of the hypoalbuminemic individuals had ketonemia exceeding 3 mmol/L compared to 19% of those with normoalbuminemia (OR: 2.12, 95% CI: 0.99–4.48, *P* = 0.051).* Conclusions*. Serum albumin concentration is inversely associated with ketosis risk in hospitalized individuals with T2DM.

## 1. Introduction

Diabetes affects an estimated 285 million individuals worldwide, most of whom have type 2 diabetes mellitus (T2DM) [1]. This number is expected to increase, with Asia becoming an epicenter of T2DM thanks to urbanization and increasingly sedentary lifestyle [2]. Apart from chronic complications, individuals with inadequately controlled disease may experience hyperosmolar hyperglycemic syndrome (HHS) and, less frequently, diabetic ketoacidosis (DKA). In HHS, residual insulin reserve minimizes ketosis, whereas substantial insulin deficiency in DKA results in ketone formation [3].

DKA involves a constellation of metabolic acidosis, ketosis, and dehydration with a mortality rate of nearly 10% [4]. Traditionally considered a unique feature of type 1 diabetes, DKA is increasingly recognized in adults with T2DM. Recent epidemiologic survey revealed that one-third of individuals hospitalized for DKA in fact harbored T2DM, which represents a sizable population given the prevalence of hospitalizations related to ketoacidosis [5]. Considering the substantial mortality risk and complications associated with DKA, identification of diabetic individuals at risk of ketosis and early preventive intervention will likely improve clinical outcome [6].

Although hemoglobin A1C (HbA1C) is an established indicator of glycemic control, it has limited correlation with disease severity during hyperglycemic crisis [7]. In contrast, several studies have validated serum albumin as a prognostic marker in severe illness. Importantly, in the context of diabetes, albumin synthesis is dependent on adequate insulin reserve [8–10]. In an outpatient setting, serum albumin concentration was found to be inversely associated with HbA1C [11], suggesting that hypoalbuminemia may reflect insulin deficiency and subsequent hyperglycemia. Essentially, serum albumin may be an indirect yet sensitive indicator of insulin secretory reserve, which influences both ketosis risk and glycemic control. Serum albumin concentration may have an advantage over conventional markers such as C-peptide, which is susceptible to interference by blood glucose level and may not closely reflect insulin secretion [12].

On the basis of these considerations, this study investigated the association between serum albumin concentration and ketosis risk in hospitalized individuals with T2DM. Furthermore, the relationship between serum albumin concentration and long-term glycemic control, as represented by HbA1C level, was examined.

## 2. Materials and Methods

### 2.1. Study Design

This was a retrospective cross-sectional study conducted at a medical center in Taiwan. Individuals hospitalized between September 2010 and August 2015 in the endocrinology ward with a principal diagnosis of hyperglycemia were screened for eligibility. Inclusion criteria were individuals exceeding 21 years of age, with a preexisting diagnosis of T2DM, and blood glucose concentration above 13.9 millimoles per liter (mmol/L) at admission. The diagnosis of diabetes was made using American Diabetes Association criteria, with fasting plasma glucose ≥ 126 milligrams per deciliter (mg/dL), 2-hour post-prandial plasma glucose ≥ 200 mg/dL, or HbA1c ≥ 6.5% [3]. Exclusion criteria encompassed individuals without measurement of serum albumin concentration, urine ketone, or HbA1C. Patients with active infection, myocardial infarction, or cerebrovascular event that may adversely affect glycemic control were also excluded. Furthermore, individuals with liver cirrhosis, malignancy, or overt proteinuria that may alter albumin production or excretion were ineligible.

Demographic and laboratory data were extracted from electronic medical records. For each subject, laboratory data during hospitalization were analyzed, while HbA1C closest to admission within the previous three months was selected. Hypoalbuminemia was defined as serum albumin concentration below 3.0 grams per deciliter (g/dL) [13]. Ketonuria was established by the presence of ketones in a urine sample. Furthermore, substantial ketonemia was defined as serum beta-hydroxybutyrate (BHB) concentration exceeding 3 mmol/L, which has been established as one of the criteria for DKA [14]. The study was approved by the ethics committee of the medical center.

### 2.2. Laboratory Methods

HbA1c was measured by ion-exchange high-performance liquid chromatography using BioRad VARIANT*™* II Turbo system. Urine ketone was determined by Beckman Coulter iRICELL2000*™* system. Serum albumin was measured by bromocresol purple method using Beckman Coulter UniCel DxC 800 Synchron*™* Clinical Systems. Serum BHB was measured by Abbot Laboratories Optium Xceed*™* system.

### 2.3. Statistical Analysis

Student's* t*-test was used to compare continuous variables and Pearson's chi-squared test for categorical variables. Cross-tabulation enabled calculation of the odds ratio (OR) and 95% confidence interval (CI). Pearson's correlation was employed to examine the relationship between clinical variables. All calculations were based on a two-sided hypothesis with *P* < 0.05 interpreted as significant. Statistical Package for the Social Sciences version 22 was used for statistical analysis (SPSS, Inc., Chicago, IL).

## 3. Results

After screening according to the abovementioned criteria, 255 individuals were included in the study. Subjects were classified according to serum albumin concentration into two groups. Individuals with serum albumin concentration above 3.0 g/dL were designated as normoalbuminemic controls, while those with serum albumin below this threshold were classified as hypoalbuminemic cases. Their demographic characteristics are shown in Table 1. Both groups were similar in age, sex, body mass index, and serum creatinine concentration.

The prevalence of ketonuria was 48% in hypoalbuminemic subjects compared to 30% in those with normoalbuminemia (OR: 2.15; 95% CI: 1.26–3.57; *P* = 0.004). Moreover, for the 156 subjects with serum BHB measurement in addition to urine ketones, 33% of individuals with hypoalbuminemia harbored ketonemia exceeding 3 mmol/L, compared to 19% of those with normoalbuminemia (OR: 2.12, 95% CI: 0.99–4.48, *P* = 0.051). These findings are presented in Table 2.

**Figure 1:**
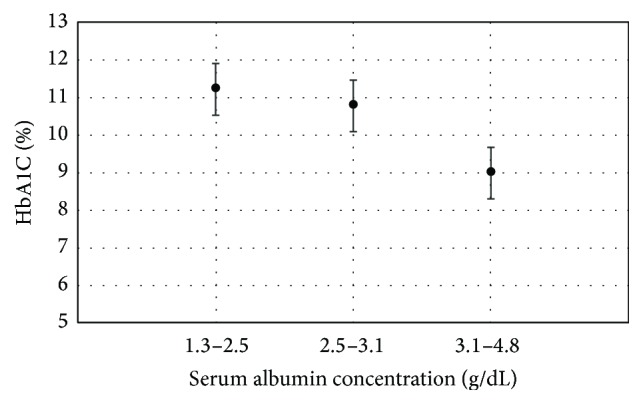


Pearson's correlation was employed to uncover the relationship between serum albumin concentration and other clinical variables, and the results are documented in Table 3. Serum albumin concentration was found to be negatively correlated with HbA1C (*r* = −0.293, *P* < 0.001). A plot of mean HbA1C to serum albumin concentration in tertiles also revealed an inverse relationship, as demonstrated in Figure 1.

## 4. Discussion

Insulin is an important regulator of albumin synthesis. This has been corroborated by impaired liver production of albumin during insulin deficiency and a 10% increase in daily albumin synthesis after insulin infusion in diabetic individuals [15]. In animal models of insulin deficiency, serum albumin concentration was normalized after three days of insulin therapy, further strengthening the link between insulin reserve and serum albumin concentration [16]. In clinical practice, an association between hypoalbuminemia and insulin deficiency may explain the increased mortality rate in hyperglycemic individuals with low serum albumin concentration [17].

In this study, diabetic individuals with low serum albumin concentration were susceptible to ketosis, as evidenced by a higher prevalence of ketonuria and a trend towards increased ketonemia in this population. Therefore, hypoalbuminemia may be a useful marker of ketosis risk, which in turn suggests underlying insulin deficiency. In this context, serum albumin concentration may indirectly reflect a diabetic individual's insulin reserve and thus serve as an indirect indicator of glycemic control. Moreover, an inverse association was observed between serum albumin concentration and HbA1C. Previous studies involving outpatients have demonstrated similar findings [11, 18]. One advantage of utilizing serum albumin as a measure of glycemic control is its shorter half-life of 21 days, which renders its serum concentration more sensitive to recent change in average blood glucose level than HbA1C [19].

Hyperglycemia exacerbates beta cell dysfunction and depletes insulin secretory reserve [20]. There is no convenient measurement of beta cell function, with several investigators using proinsulin to insulin ratio for research purposes [21]. However, proinsulin measurement is unavailable to most hospital laboratories, and the proinsulin to insulin ratio will likely be altered by exogenous insulin used to treat hyperglycemia. Furthermore, current guidelines for hyperglycemic crisis target the resolution of hyperglycemia and metabolic acidosis. In practice, numerous recuperated patients experience recurrent ketosis after withdrawing insulin therapy, implying that impaired insulin secretion persists after clinical recovery. Serum albumin, which is commonly available to hospital laboratories and indicative of an individual's insulin secretory reserve, may assist clinicians in determining the duration of insulin therapy.

Several other implications arise from the observations in this study. First, diabetic inpatients with hypoalbuminemia are very likely to have depleted insulin reserve and are likely to require early initiation of insulin therapy to prevent ketosis. Furthermore, serum albumin concentration may enable stratification of hyperglycemic individuals with respect to ketosis risk and prognosis. In fact, serum albumin has been validated as a measure of disease severity in the Acute Physiology and Chronic Health Evaluation scoring system [22]. Finally, along with HbA1C, serum albumin may serve as an adjunctive measure of glycemic control which better reflects recent change in average blood glucose level.

This study benefits from a population of individuals who were hospitalized principally for acute hyperglycemia, with the exclusion of factors such as active infection, liver cirrhosis, malignancy, and overt proteinuria that may adversely affect glycemic control. To enable accurate interpretation of the results, only laboratory data during the hospitalization period were analyzed, with the exception of HbA1C, for which measurements within the three months prior to admission were selected. Finally, demographic characteristics were similar between cases and controls, which minimized potential confounding effects.

Several limitations arise from the retrospective design. Because serum albumin concentration was more frequently tested in malnourished or severely ill individuals who were suspected of hypoalbuminemia, this may have led to an inherent selection bias. Furthermore, treatment modalities prior to hospitalization that affect ketosis risk and glycemic control, such as exogenous insulin, were not taken into account. Finally, although serum BHB is a more specific marker of ketosis, it was only available for a subset of individuals in this study.

## 5. Conclusions

In conclusion, serum albumin concentration is inversely associated with ketosis risk in hospitalized individuals with T2DM. Diabetic individuals with low serum albumin concentration are at risk of ketosis and may require early initiation of insulin therapy to prevent complications. Furthermore, subjects with hypoalbuminemia have higher HbA1C levels compared to those with normoalbuminemia. Serum albumin concentration potentially mirrors an individual's insulin secretory reserve, which subsequently influences glycemic control and ketosis risk. Serum albumin concentration is a promising prognostic marker in hospitalized diabetic individuals with acute hyperglycemia.

## Figures and Tables

**Table 1:** Demographic characteristics.

	Albumin < 3.0 g/dL (*n* = 151)	Albumin ≥ 3.0 g/dL (*n* = 104)	*P* value
Age (years)	68 ± 1.4	69 ± 1.5	0.97^a^
Sex			
Female (%)	52.3	51.0	0.83^b^
Male (%)	47.7	49.0	
BMI^c^ (kg/m^2^)	22 ± 0.4	23 ± 0.4	0.94^a^
SCr^d^ (mg/dL)	1.91 ± 0.18	2.16 ± 0.19	0.37^a^

^a^Student's *t*-test.

^b^Pearson's chi-squared test.

^c^Body mass index (BMI).

^d^Serum creatinine (SCr).

**Table 2:** Assessment of ketosis risk.

	Albumin < 3.0 g/dL (%)	Albumin ≥ 3.0 g/dL (%)	OR^a^	95% CI^b^	*P* value^c^
Ketonuria	72 (48)	31 (30)	2.15	1.26–3.57	0.004^*∗*^
No ketonuria	79 (52)	73 (70)			

	*n* = 151	*n* = 104			

Serum BHB^d^ > 3 mmol/L	28 (33)	13 (19)	2.12	0.99–4.48	0.051
Serum BHB ≦ 3 mmol/L	58 (67)	57 (81)			

	*n* = 86	*n* = 70			

^a^Odds ratio (OR).

^b^Confidence interval (CI).

^c^Pearson's chi-squared test.

^d^Beta-hydroxybutyrate (BHB).

^*∗*^Statistical significance (*P* < 0.05).

**Table 3:** Relationship between serum albumin concentration and clinical variables.

	*r* ^a^	*P* value
HbA1C	−0.293	<0.001^*∗*^
BMI	0.093	0.14
Creatinine	−0.008	0.90
Age	−0.018	0.77

^a^Pearson's correlation.

^*∗*^Statistical significance (*P* < 0.05).
